# Global Thrombosis Test: Occlusion by Coagulation or SIPA?

**DOI:** 10.1055/s-0041-1732341

**Published:** 2021-09-19

**Authors:** Viviana Clavería, Patricia J. Yang, Michael T. Griffin, David N. Ku

**Affiliations:** 1 GWW School of Mechanical Engineering, Institute for Bioengineering and Biosciences, Georgia Institute of Technology, Atlanta, Georgia, United States

**Keywords:** GTT thrombosis, point-of-care, coagulation, SIPA, shear rate

## Abstract

The global thrombosis test (GTT) is a point of care device that tests thrombotic and thrombolytic status. The device exposes whole blood flow to a combination of both high and low shear stress past and between ball bearings potentially causing thrombin and fibrin formation. The question arises as to whether thrombosis in the GTT is dominated by coagulation-triggered red clot or high shear-induced white clot. We investigated the nature of the thrombus formed in the GTT, the device efficacy, human factors use, and limitations. The GTT formed clots that were histologically fibrin-rich with trapped red blood cells. The occlusion time (OT) was more consistent with coagulation than high shear white clot and was strongly lengthened by heparin and citrate, two common anticoagulants. The clot was lysed by tissue plasminogen activator (tPA), also consistent with a fibrin-rich red clot. Changing the bead to a collagen-coated surface and eliminating the low shear zone between the beads induced a rapid OT consistent with a platelet-rich thrombus that was relatively resistant to heparin or tPA. The evidence points to the GTT as occluding primarily due to fibrin-rich red clot from coagulation rather than high shear platelet aggregation and occlusion associated with arterial thrombosis.

## Introduction

The global thrombosis test (GTT, Montrose Diagnostics, London, United Kingdom) is described as the first physiologically relevant point-of-care test to assess the risk of thrombosis or bleeding, or to monitor antiplatelet medication without citrate. 1 2 GTT explains the flow scenario through the device as inducing an initial high shear stress stimulus followed by a low shear stress portion using a double ball-bearing system located inside a conical test tube. Each bead has gaps between its surface and the inner tube wall. When blood is added to the tube, it flows through the gaps by the ball bearings, into large spaces above and between the beads, and the droplets are collected in a reservoir.

Occlusive thrombus can be formed along a continuum that depends on a variety of factors and flow conditions. Virchow in 1856, described a triad to form a coagulation-based red thrombus. The triad consist of stasis or low shear rate conditions, nonendothelial surface, and hypercoagulable blood that is required for thrombosis. 3 This triad stimulates the coagulation cascade by the intrinsic pathway to form fibrin with trapped red blood cells (35–60%), sometimes called a red clot because of its appearance. 3 4 5 6 7 Coagulation is strongly blocked by the anticoagulants heparin and citrate. Dissolution of these fibrin-rich red clots could be triggered by tissue plasminogen activator (tPA) to break down fibrin.

In contrast, occlusive arterial thrombi, that form under high shear rate conditions, can form over collagen exposed after plaque rupture to capture von Willebrand factor (vWF) that, in turn, aggregates platelets to form a white clot. 8 9 In the case of arterial thrombosis, a stenotic atherosclerotic plaque is the responsible for creating pathologically high wall shear rates ranging from 5,000 to 100,000 1/s, much higher than the typical wall shear rate of <1,000 1/s found in normal arteries. 10 11 When a plaque cap ruptures, prothrombogenic collagen from the extracellular matrix is exposed. The exposed collagen surface binds vWF, and subsequent platelet adhesion and shear-induced platelet aggregation (SIPA) occurs. The formation of an occlusive white clot occurs in less than 5 minutes for microfluidic dimensions. Histology studies estimate white clots to be approximately 50 to 80% platelets by volume, 12 with smaller amounts of fibrin. These two red and white thrombi are morphologically different and created by different factors, so can be distinguished experimentally by different methods, including Carstairs staining 13 where different components react giving different colors.

Platelet function tests can be designed to measure blood samples for their propensity to form thrombi by these two mechanisms, since venous thrombi are associated with low shear and arterial thrombi are formed under high shear conditions. GTT, as a point-of-care test, purports to be “the first, pathologically relevant, point-of-care test of thrombotic and thrombolytic status.” 2 It has been suggested that shear activated platelet-derived procoagulant activity plays a crucial role on thrombi formation, 14 15 but no experiments have been reported to reveal the nature of the thrombi formed within the test section of the GTT. Beyond the formation of occlusion in the device, the GTT may detect endogenous thrombolytic activity, which may be another major determinant of hemostasis. 16 Correlations between “lysis time” (LT) values and major adverse cardiovascular events (MACEs) have been observed, but not between “occlusion time” (OT) and MACE. 17

The GTT flow system is a simple and clever tool that appears to have both low and high shear zones. We investigate whether the GTT creates occlusions (OT) from the low shear coagulation or high shear platelets, and if LT represents fibrinolysis with restoration of blood flow. We then modify the GTT to create a different type of thrombus.

## Materials and Methods

To evaluate the type of thrombi formed in the GTT, we evaluated the histological appearance of formed thrombi in situ by Carstairs staining, 13 compared OT against expected clotting times from SIPA versus coagulation, sensitivity of OT to anticoagulants, and reaction of the system to tPA (Sigma Aldrich, United States). Carstairs staining clearly differentiates coagulation clots as red, different from SIPA clots that are blue. 12 13 18 We then modified the surface characteristics and shear rate zones in an attempt to identify alternative mechanisms of thrombosis. This series of tests was used to distinguish thrombosis in the GTT as being primarily from coagulation or SIPA.

The GTT device has a main unit where all the electronic equipment is located. The measured quantities of the tests are stored on an SD card and the results displayed on a screen on the front of the device. A second part consists of a disposable cartridge that consists of a test tube with a conical taper at the bottom trapping two beads aligned vertically in series. Gaps are formed in between the spherical beads surface and the molded inner wall of the test tube. The device used in this study is the latest GTT-3 model, that could be operated as GTT-2 and GTT-3. GTT-2 records OT and LT, while GTT-3 can additionally assess “thrombus stability” time and rate of thrombolysis by applying external pressure. Unless otherwise stated, we operated our instrument in the GTT-2 mode where blood flow was driven through the test tube by gravity.

**Fig. 1:**
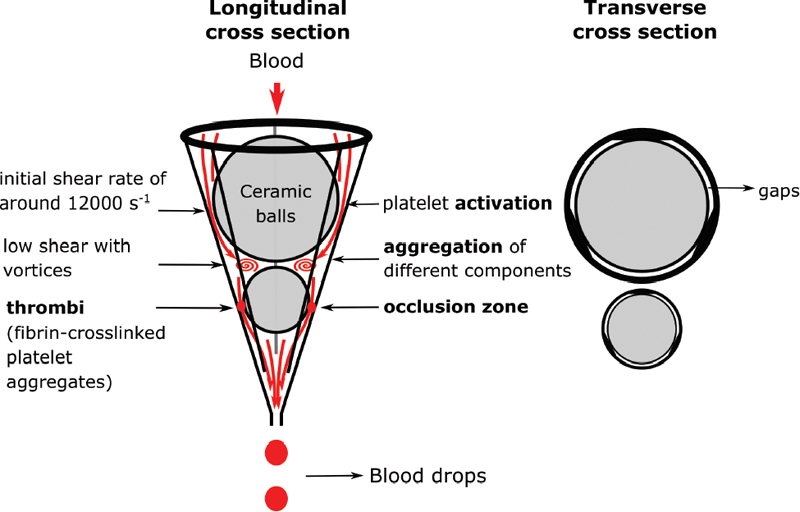
Schematic of the GTT cartridge. Blood is injected at the top of the GTT test tube and flows passing the gaps of two ceramic beads. Occlusion happens due to several stages of activation and aggregation. 1 GTT, global thrombosis test; OT, occlusion time.

As thrombus is formed, blood flow is gradually reduced. The instrument detects the time interval, *d,* between two consecutive blood drops falling into a reservoir by a light sensor ( Fig. 1 ). *d* increases with time as the flow rate decreases. When *d* ≥15 seconds the device reports the elapsed time that is displayed in seconds as OT. OT has a maximum value of 900 seconds. After OT, a period follows (typically set to 300 seconds described by the manufacturer as “thrombi stabilization period”). The first drop of blood detected by the photosensor after this “stabilization period” indicates the beginning of spontaneous thrombolysis, according to the manufacturer. The total time from the beginning of the test until this point is called T1. The lysis time (LT) is defined as LT = T1–OT. If lysis does not occur until 6,000 seconds after OT (LT cutoff time), “no lysis” is displayed. 1

We first examined the beads, and the test section dimensions and material. The manufacturer does not provide details as to the composition or surface coating on the beads. We scanned the beads in the tube by using microcomputerized tomography scan (micro-CT) (Scanco uCT50) to visualize the size and number of gaps in between the beads and the test tube.

To run the device, 4 mL of blood from a syringe is injected into the test tube by hand within 3 to 5 seconds. Since the procedure was manually performed, we scrutinized if results were user dependent and evaluated their variability. Once the blood was injected, it flows by gravity passing through the gaps. The time interval between two drops is recorded onto the SD card and used to quantify the OT and LT of the sample with the standard parameters set up in the device for the mode GTT-2. The results are additionally displayed on the front screen of the device and the raw data stored on the removable SD card.

Shear rate in the space between the gaps and the inner wall test section was estimated by approximating the gap to be a rectangle with a length *L* (length of the gap arc) and width *w* . Estimation of shear rate in the gap was done using the following equation for 2D channel flow



where the flow rate ( *Q* ) was estimated experimentally by measuring the mass rate assuming a blood density of 1.06 g/mL. 19 The flow shear rate generated in the gaps was stated by the manufacturer to be 4,000 to 12,000 1/s without further evidence. Using our estimated shear rate from Eq. (1) and the initial geometrical characteristics on the channels, we used the predictive model of SIPA thrombus formation developed by Mehrabadi et al 20 to predict the OT from SIPA in the gap.

Once clots were formed and OT reached, histology of retrieved clots allowed us to identify the composition of thrombus formed in the test tube. We harvested the clots at and after OT and used Carstairs' stain 13 21 22 to identify erythrocyte, platelet, and fibrin content.

Blood was drawn from healthy volunteers at the Georgia Tech Stamps Health Laboratory (IRB Protocol Number: H18238) using 21-gauge butterfly needles and syringes, without or with anticoagulants. The preloaded anticoagulants used were heparin sodium or sodium citrate (Sigma Aldrich, United States). Blood samples were collected under the following conditions: 4 mL of fresh blood without any anticoagulant was tested within 15 seconds of drawing; 20 mL of blood heparinized at 3.5 USP units/mL; or 18 mL of blood treated with 2 mL citrate solution (3.2 wt% in 0.9% saline). The anticoagulated blood was stored at room temperature on a shaker before testing within 3 hours. OT and LT were measured for the blood samples. For separate samples, LT was measured for blood treated with PBS enriched with 50 nM of tPA (study) or phosphate buffered saline (PBS, Sigma Aldrich, United States) only as control.

We hypothesized that to produce SIPA, there should be a surface for vWF and platelets to attach. 18 23 24 25 Fibrillar collagen is a known, potent surface for SIPA. 26 We modified the original GTT device replacing the two ceramic beads by one glass bead of 4 mm diameter (soda-lime glass, Sigma Aldrich) that we coated with collagen (Fibrillar type I collagen, Sigma Aldrich, United States) as a prothrombotic surface. We called the GTT with this modification included, “modified GTT” or mGTT. Finally, we compared the OT and LT in the mGTT to our microfluidic thrombosis assay (MTA) representing an arterial stenosis as described by Griffin et al. 27 The MTA incorporates the major factors for arterial thrombosis including fibrillar collagen and a well-defined high shear zone to create a predictive model of SIPA that has been validated against clinical arterial thrombosis.

## Results

### GTT

#### Occlusion Time

OT obtained for fresh human blood in our GTT device was 526 ± 188 seconds with an intra-assay variation of 15% ( *n* = 7) and an inter-individual variation of 36% ( *n* = 7). The mean value for OT is consistent with several reference articles, but not to others ( Table 2 ).

**Table 1:** The bead diameter ( *D* ), arc, lengths ( *L* ), width ( *w* ), area ( *A* ), flow rate ( *Q* ), and shear rate (γ) in each gap around the 4- and 3-mm beads for a total flow rate of 3.8 (mL/s) estimated from the experimentally measured total mass rate of 4 mg/s

*D* (mm)	Arc (°)	*L* (mm)	*w* (mm)	*A* (mm ^2^ )	*Q* (mL/s)	(1/s)
4	113	3.94	60	0.24	2.58	1,100
4	101	3.53	10	0.04	0.38	6,500
4	110	3.84	20	0.08	0.84	3,300
3	102	2.67	31	0.08 a	2.30	5,400
3	65	1.70	28	0.05	1.33	6,000
3	13	0.34	18	0.01	0.17	9,300

Note: We assumed a blood density of 1.06 g/mL.

a Area is shown in Fig. 10 as an example.

**Table 2:** The occlusion time (OT) in the literature for different bead materials

Mean (s)	Std (s)	Inter-individual CV (%)	Intra-assay CV (%)	Intra-day CV (%)	No. of gaps	Bead material	Publication year	Ref
185.0	9.2 b	22.9 c			2	steel	2003	36
205.8	12.7 b	20.9 c			2	steel	2003	36
154.8	64.7	41.8 c			NA	NA	2006	37
391	40	10.2 c	12	6.2 f	NA	NA	2009	38
379	30	7.9 c	12	6.2 f	NA	NA	2009	38
441	32	7.3 c	12	6.2 f	NA	NA	2009	38
284.9	92	32.3 c			4	steel	2010	30
378.0	96	25.4 c		6.2 e	4	metal	2010	16
464.5 a					NA	steel	2012	31
495 a					NA	steel	2014	32
481		27			4	ceramic	2014	14
419 a				6 d	4	metal	2014	34
524.9	17	3.2 c			4	ceramic	2015	33
**526**	**188**	**36**	**15**		**3**	**Ceramic**	**2019**	**Our study**

Abbreviation: NA, not available value.

a indicates median values instead of mean.

b indicates standard error instead of standard deviation.

c The inter-individual CV (coefficient of variation) is estimated based on mean, and standard deviation or sample size, and standard error in the corresponding literature.

d CV obtained testing a healthy volunteer four times, at 24-hour intervals.

e CV was assessed by testing 1 healthy volunteer 10 times over a period of 4 weeks, under similar conditions, and by testing 10 volunteers twice, at 48-hour intervals.

f One healthy female subject was tested twice a week for 4 weeks by the same technician, under the same conditions, always in the afternoon.

#### Clot Histology

**Fig. 2:**
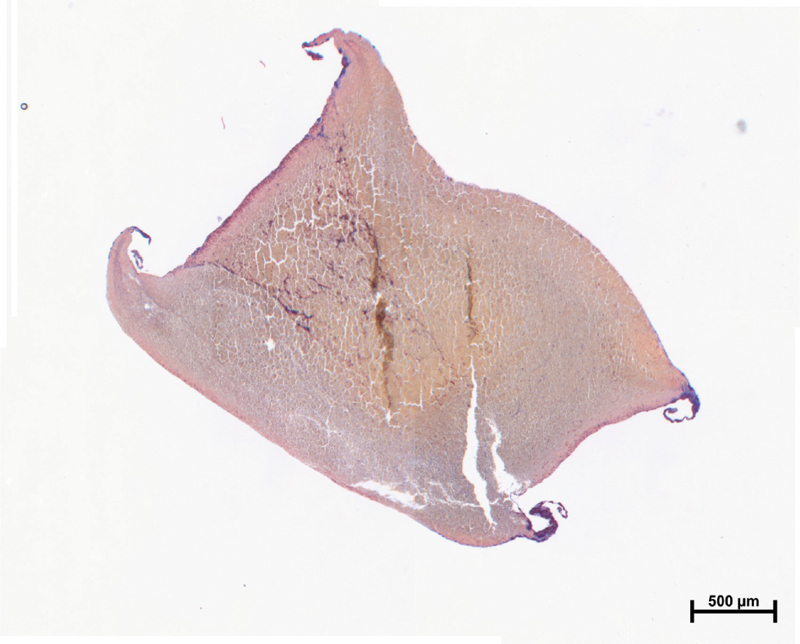
Histology of the GTT clot using Carstairs' stain. Red indicates fibrin and blue platelets. GTT, global thrombosis test.

Although blood clots were expected to appear at the gap between beads and the wall, 28 we did not see clots at this location at the time of OT. It could be that clots were too small to be visible to the naked eye. Clots were clearly visible after OT, between 500 and 1,000 seconds. To observe the time course of clot growth, we tested identical blood samples and rinsed the tube with PBS at different times after the OT to remove all blood components except the ones forming the clot. The blood clot was not visible at an OT of 415 seconds but visible at 800 seconds between both 3 and 4 mm ceramic beads. The clot attached to beads was located not only between two beads but also above the 4 mm bead. We harvested those clots and used a Carstairs' stain to visualize fibrin, red blood cells, and platelets. The histology of the GTT clot was predominately fibrin (red) without platelets ( Fig. 2 ). Some light blue tinge appears close to all artificial surfaces. Dark blue concentrations could be observed at the extensions (presumably the gaps), with more platelets at the 3 mm bead. We could not distinguish from histology whether flow occlusion was predominantly from clots at the gaps or from the large clot forming between the beads.

#### Lysis Time

**Fig. 3:**
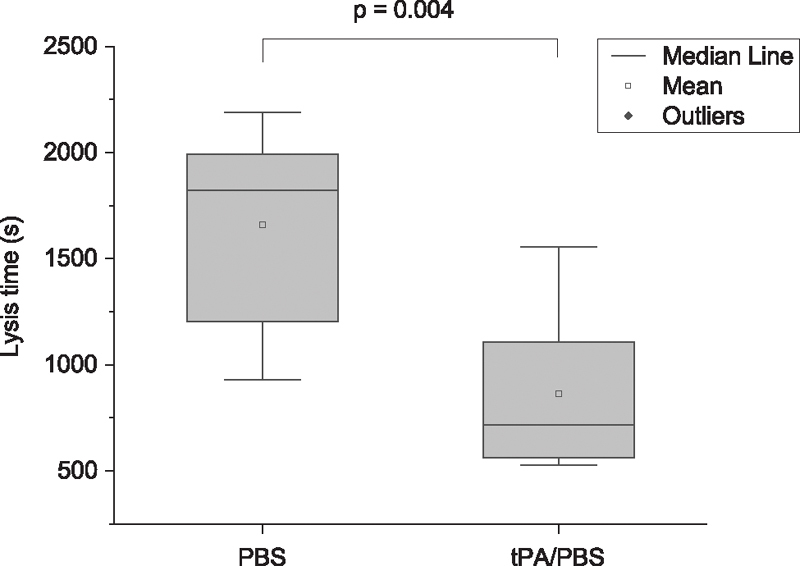
Box charts of LT obtained from the blood samples treated with PBS or tPA in PBS through the GTT device after clot formation. GTT, global thrombosis test; PBS, phosphate buffered saline; tPA, tissue plasminogen activator.

LT was obtained for fresh blood and with added tPA. For the standard GTT procedure, the average LT of 1,660 ± 495 seconds corresponding to “normal spontaneous thrombolytic activity” reported in the GTT user manual (LT <2,000 seconds). In an effort to characterize the composition of the occluding clot, the blood samples were treated with tPA in PBS at the beginning of the experiment, before measuring OT. The tPA group was compared with a control group with added PBS solution so that dilutional effects were factored into the experiments. Blood from the same individuals collected at the same time were used for both groups. In the tPA group, LT was reduced to 865 ± 404 seconds which is approximately half of the time seen in the control group ( Fig. 3 ). The paired *t* -test reveals the significance of the difference between the two groups ( *p* = 0.004, *n* = 6, including two measurements for each individual from three individuals). The reduction of LT is consistent with a previous GTT study administering a similar concentration of tPA. 28

#### Heparin

**Fig. 4:**
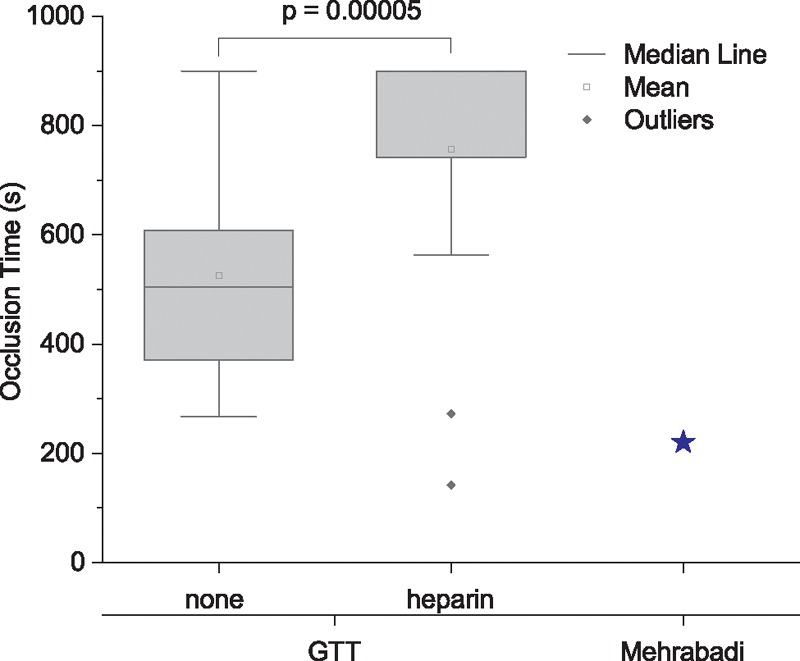
Box charts of OT obtained for GTT for fresh blood and blood with the anticoagulant heparin. The star in the plot is the predicted value for OT obtained for SIPA using the model developed by Mehrabadi et al for SIPA in heparin. 20 GTT, global thrombosis test; OT, occlusion time; SIPA, shear-induced platelet aggregation.

To further explore the properties of clots in the GTT, we used additives that differentially affect the formation of coagulation versus shear-induced platelet aggregation (SIPA). Heparin strongly inhibits coagulation and the formation of fibrin so that we might expect increases in OT if clots are fibrin rich. In the GTT device, fresh blood without heparin occludes with an average OT of 527 ± 204 seconds; whereas, heparinized blood has an average OT of 847 ± 106 seconds as a lower bound ( *p* = 0.00005, *n* = 12 measurements, including two measurements from six individuals) while nine out of 12 measurements never reached the OT beyond the limit of GTT (>900 seconds ( Fig. 4 ). This strong effect by heparin suggests that the main cause for occlusion in the GTT is coagulation. For comparison, the OT for fresh blood of 527 ± 204 seconds is almost double the predicted OT of 222 seconds from SIPA ( Fig. 4 ). 20

Thus, the exposure of blood to the ceramic contact surface, OT being longer than SIPA, the histological appearance of a predominantly fibrin-rich red clot, the short LT by tPA, and the lengthening of OT by heparin all point to thrombotic occlusion in GTT as strongly dominated by coagulation.

### Modified GTT (mGTT)

We next explored whether the system could be modified to create high shear platelet-vWF rich thrombi instead. SIPA thrombi form in the presence of three factors: (1) vWF and platelets in whole blood; (2) high shear rates; and (3) fibrillar collagen surface. 18 23 24 25 The first two factors are already present in the GTT. Previous work has demonstrated that fibrillar collagen can be deposited on glass. 26 Consequently, we modified the original GTT (mGTT) to promote the creation of platelet-rich clots at high shear rates by replacing the two ceramic beads with a single glass bead of 4 mm in diameter, coated with fibrillar type I collagen. With a single bead, we also avoid the low shear zone in between the beads, where the clots formed in the GTT. The blood flow through the gap of the coated 4 mm bead was 16 ± 1 mg/s, corresponding to the measured volumetric flow rate of *Q* = 15 ìl/s. The resulting initial shear rate under these conditions was 6,700 1/s, two times higher than the average of 3,500 1/s reached in the gap of the original 4 mm ceramic bead of the GTT device.

#### Occlusion Time

**Fig. 5:**
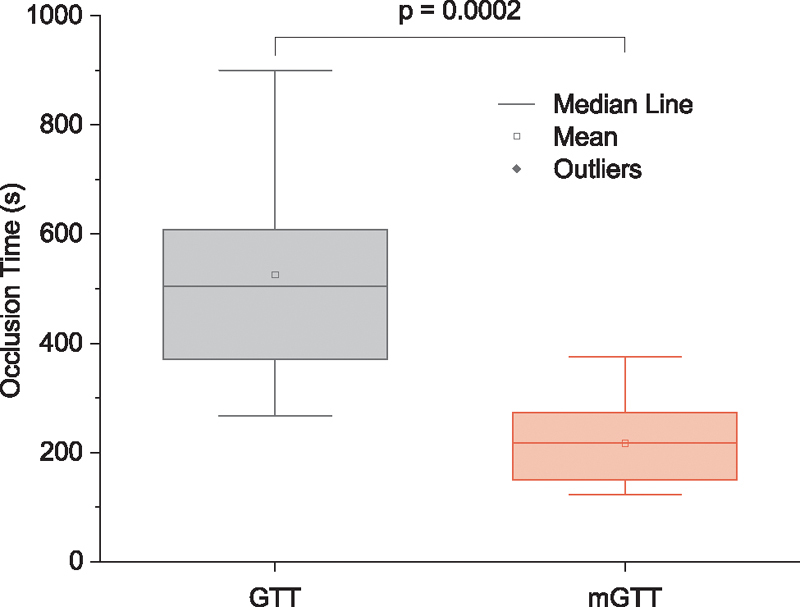
Box charts of OT obtained flowing fresh blood through GTT and mGTT. mGTT adds a collagen surface, higher shear stress, and eliminates the low shear zone between the beads. mGTT, modified global thrombosis test; OT, occlusion time.

We tested mGTT using fresh blood from the same seven healthy subjects tested on the GTT system. The average OT in mGTT was 217 ± 71 seconds about half of that obtained in GTT (OT = 526 ± 188 seconds *p* = 0.0002, *n* = 14 including two measurements from seven individuals) ( Fig. 5 ). Notice that variability decreases approximately 10% in the mGTT compared with the GTT device. The OT of 217 ± 71 seconds obtained by the mGTT is also close to the empirical prediction of 222 seconds for high shear SIPA developed by Mehrabadi et al. 20

#### Clot Histology

**Fig. 6:**
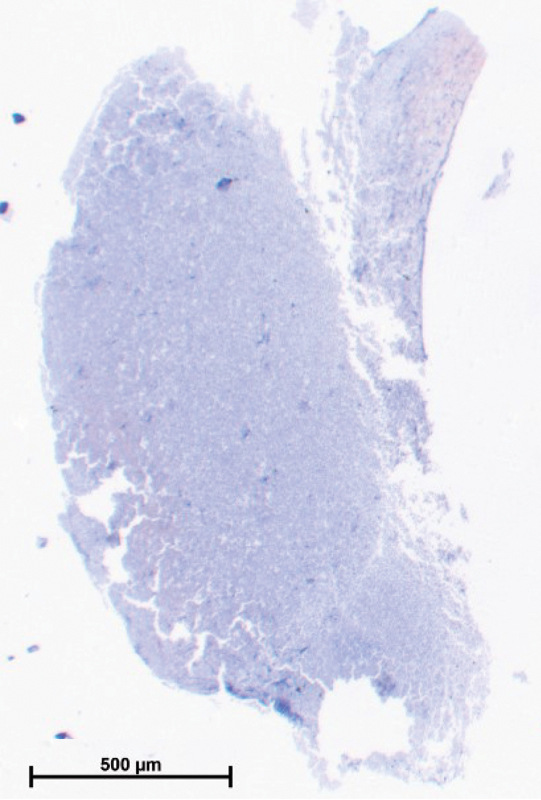
Histology of the mGTT clot using Carstairs' stain. The thrombus is composed primarily of platelets are stained blue, and the tinge of red indicates small amounts of fibrin. Few RBC are present in this white clot. mGTT, modified global thrombosis test; RBC, red blood cell.

Histology of thrombi obtained in the mGTT system revealed platelet-rich clots as visualized in blue with Carstairs staining of clots harvested at approximately 200 and 400 seconds. The mGTT clots did show the presence of some fibrin (red) (see Fig. 6 ). At approximately 200 seconds the blood clot is barely visible as a dot on the bead, which then grows as a tail by 400 seconds.

#### Lysis time

We measured LT for mGTT in the same way done for GTT by enriching the blood sample with tPA. For comparison purposes, we used blood from the same individual for the tPA group (blood with tPA/PBS solution) and the control group (blood with PBS solution). In the control group ( *n* = 3), the average LT was 4375 ± 1660 seconds while with tPA, the value obtained was 2110 ± 2116 seconds ( *p* = 0.12, *n* = 6 including 2 measurements from 3 individuals). Note, the values for LT in the mGTT are more than the double that produced in GTT.

#### Heparin and Citrate

**Fig. 7:**
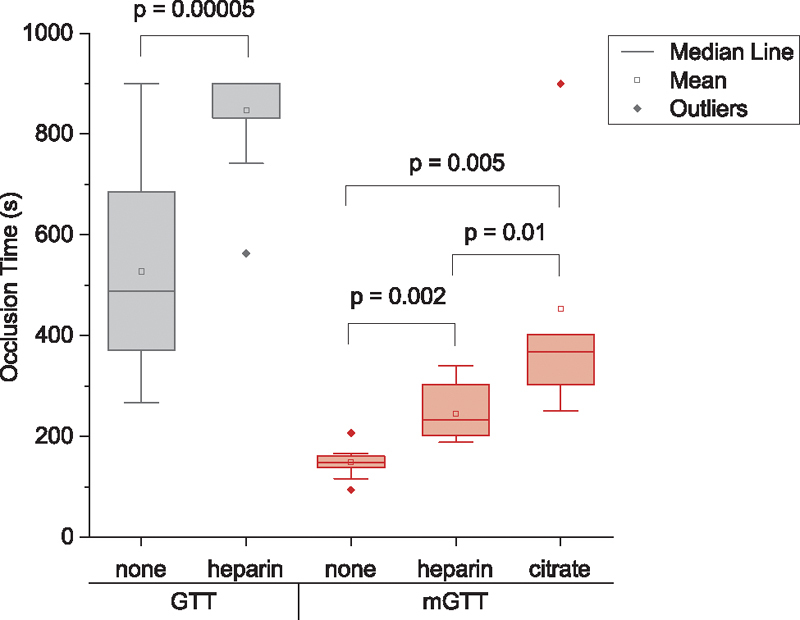
Box charts of OT obtained for GTT and mGTT for non-anticoagulated blood (“none” in the graph) and anticoagulated blood with heparin for both GTT and mGTT and citrate for the mGTT. mGTT, modified global thrombosis test; OT, occlusion time.

We tested the effects of anticoagulants on the OT for the mGTT. Heparin was expected to prevent fibrin formation, so the effect of heparin may inform the amount of fibrin contributing to the formation of the clot. In the mGTT system, heparinized blood at 3.5 USP units/mL increased the OT to 244 ± 53 seconds compared with 148 ± 30 seconds for nonheparinized blood ( *p* = 0.002, *n* = 10 including 2 measurements from 5 individuals), suggesting that there is some contribution of fibrin in the SIPA thrombi formation within the GTT ( Fig. 7 ). Notice that the OT in the mGTT was smaller than the GTT even with heparin, so occlusion was happening faster in mGTT than in GTT. The variance is also lower in the mGTT than the GTT for fresh blood measurements. We also compared the OT from GTT and mGTT results to an equivalent OT obtained using an MTA 27 with heparinized blood. The equivalent OT in the MTA was 189 ± 50 seconds ( *n* = 44 measurements in total. 8 measurements for each individual for five individuals plus another four measurements of one extra individual). *p* = 0.01 between mGTT OT of 244 ± 53 seconds for heparinized blood compared with MTA OT. *p* -Value was obtained under unpaired *t* -test without assuming equal variance. OT measured on the MTA is also much shorter than the OT obtained on the GTT of 847 ± 106 seconds for heparinized blood ( *p* = 5.3 × 10 ^−11^ under unpaired *t* -test without assuming equal variance).

To further explore the stability of clots formed in the mGTT, we used blood enriched with citrate, a known anticoagulant used for platelet studies that is reported to prevent occlusion in the GTT. 28 The OT in the mGTT using citrate, yielded an OT of 453 ± 240 seconds ( Fig. 7 ), similar to the OT for GTT using fresh blood.

#### Use of Noncoated Glass Beads

**Fig. 8:**
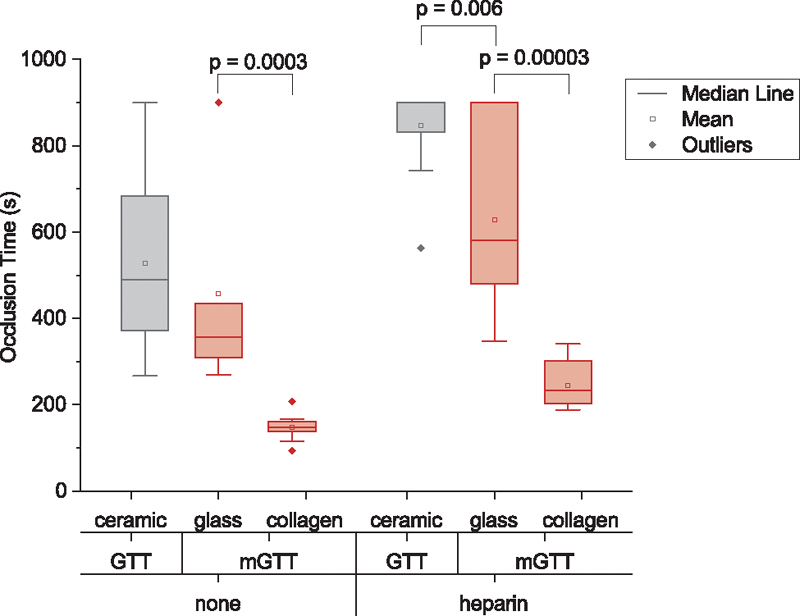
Box charts of OT obtained for three different bead surfaces: ceramic, glass, and collagen coated. GTT and mGTT were tested with blood samples without anticoagulants ( *none* in graph) and with heparin as an anticoagulant. OT, occlusion time.

We have previously shown that the collagen-coating is critical in the formation of SIPA occlusions. 18 23 24 25 To confirm the same effect in the GTT, we tested the difference in OT between the collagen-coated and a clean glass bead in the mGTT device using blood from the same population without an anticoagulant or with heparin. Clean glass beads (without coating) led to an OT of 457 ± 240 seconds which is 3 times higher than that of 148 ± 30 seconds obtained for collagen-coated glass beads ( *p* = 0.0003, *n* = 10, including 2 measurements from 5 individuals, see Fig. 8 ). We did not find a significant difference in OT using a single native glass bead or two ceramic beads in GTT. Adding heparin to the blood sample induced a lengthening for mGTT with a clean glass bead that produced an OT of 628 ± 215 seconds compared with 244 ± 53 seconds when the glass surface was coated with collagen ( *p* = 0.00003, *n* = 10, including two measurements from five individuals).

The surface properties of the bead also affect the OT using blood samples with heparin ( *p* = 0.006 in Fig. 8 between ceramic and glass surfaces in the GTT device). The ceramic beads in GTT only have 3 out of 12 samples with detectable OT within 900 seconds (OT = 847 ± 106 seconds *n* = 12, including two measurements from six individuals), but the clean glass bead in mGTT leads to occlusion for 7 out 10 sample (OT = 628 ± 215 seconds *n* = 10, including two measurements from five individuals).

#### Beads and Gaps

**Fig. 9:**
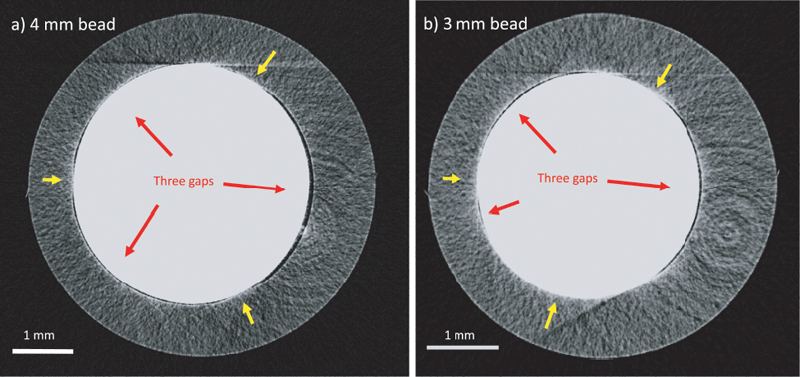
Micro-CT scan of a 4 mm and 3 mm beads ( **a** ) and ( **b** ), respectively. The gaps are the dark lines in between the ceramic bead ( *in white* ) and the test tube ( *gray* ) and indicated by red arrows. Yellow arrows indicate the places where the bead and the test tube touch their surfaces.

Thrombosis requires a nucleating surface and the material can significantly alter its thrombogenicity. 29 The GTT has been described as having beads made of steel 30 31 32 or ceramic. 14 31 The test tubes supplied to us in early 2019 had beads that were white ceramic of 4 and 3 mm in diameter. We attempted to verify the number of gaps and the gap size between the beads and the inner wall surface of the test tube by micro-CT scan shown in Fig. 9 . The gaps were much thinner than published drawings, measuring approximately 40 microns. Three unevenly sized gaps were seen by micro-CT with the 4- and 3-mm beads. As the beads are in a conical section, small changes in angulation of the tube may cause distortions in the visualization of the gaps and also in the wall thickness. The ambiguity in gap size made calculation of the true shear rates difficult.

#### Shear Rate Through the Gaps

Once blood was injected, it flowed through the gaps between beads and inner wall of the test tube at an average measured mass rate of 4 ± 2 mg/s ( *n* = eight measurements), corresponding to a flow rate Q = 3.8 μL/s for a blood density = 1.06 g/mL. Using the images from the micro-CT scan, we measured the total arc angle of gaps, *α* , to estimate the length of the gap, *L* , using Eq. (2) .



**Fig. 10:**
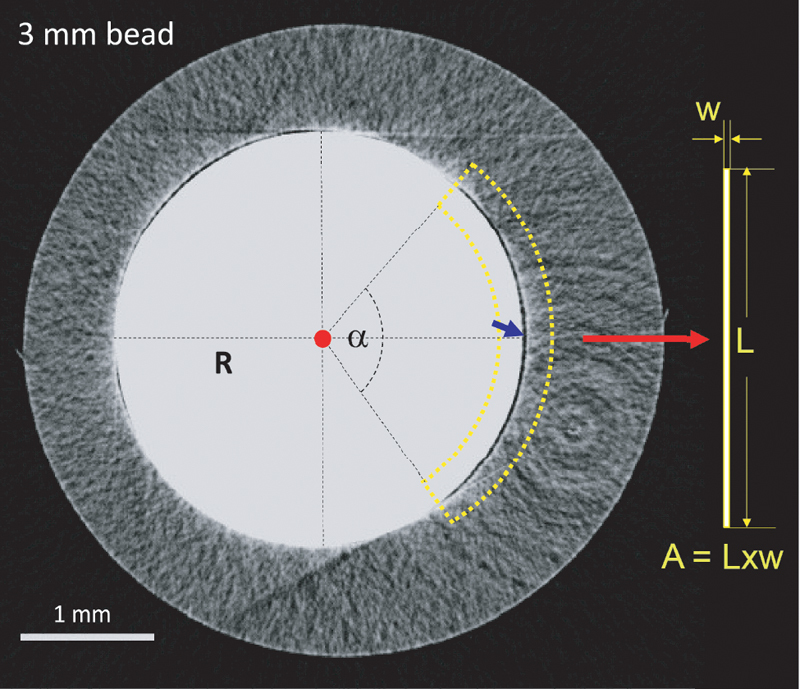
The gap geometry is approximate to be a rectangle of length *L* using Eq. (2), where *α* is the gap arc angle and *R* the bead radius, as shown as an example in the image, and width *w* , which is obtained from roughly the middle of the gap indicated by the blue arrow in the image. The gap area is then calculated as *A* = *L* × *w* .

The width of gaps ( *w* ) is approximated by the width of the widest section among gaps. The 4-mm bead has three gaps with an arc angle of 113, 101, and 110 degrees. The 3-mm bead has three gaps with arc angles of 102, 65, and 13 degrees by micro-CT ( Table 1 ). We do not know if other test tubes are manufactured differently. The equivalent gap's length ( *L* ) is multiplied by the gap's width ( *w* ) to obtain the gap's transversal area ( *A* ) ( Fig. 10 ).

The flow rate ( *Q* ) passing by each gap will be proportional to its area. The shear rate,  , is then calculated for each gap using the Eq. (1). The resulting shear rates produced in each gap around the 4- and 3-mm beads are summarized in Table 1 . The configuration of the gaps in GTT creates different shear rate zones.

#### Injection Rate

To our surprise, the results in OT were quite dependent on manual injection rates, pressures, air inside the syringe while injecting blood, and resultant bubble formation in the tube. Initially, we had poor reproducibility following the manufacturer's instructions and discussion by email. Eventually, we obtained more consistent results after >50 injections of blood in tubes placed on a rack where we could visualize the blood after injection. The injection rate and angle affected the ability of blood to not flow at all or flow fast through the exit. In our hands, the rate of injection and time of injection could be easily varied to yield different results. After weeks of trials, we were able to settle on a consistent technique of injecting the blood over 4 second each time, just off axis.

#### Data Readout

**Fig. 11:**
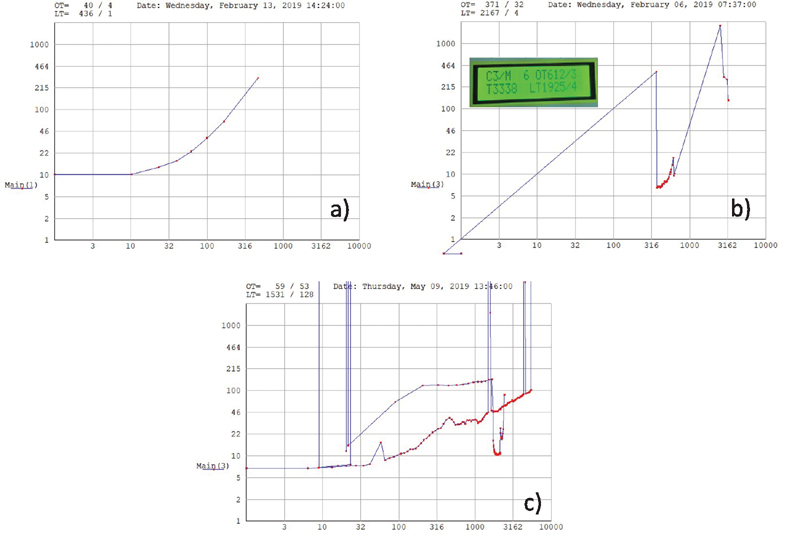
Examples of the readout from the SD stored data versus the data shown in the display. The *x* axis of the graphs should be cumulative running time, and the *y* axis should display the time, *d,* between two consecutive drops. In ( **a** ) the stored values increase smoothly and monotonically, as it is expected from the measurements. In contrast, other traces are shown in ( **b** ) and ( **c** ), where the stored values had jumps back and forth in time ( *x* and *y* axis), even showing *negative* time points. In ( **b** ), the displayed data was OT 612 and LT 1925, but the readout from the SD card is OT 317 and LT 2167. Also, there is a long time-delay for the first drop to register. The x-axis records time in a nonstandard unit that is not in seconds. In ( **c** ), it is difficult to understand this output. LT, lysis time; OT, occlusion time; SD, standard deviation.

We found that there were inconsistent values of OT and LT between the values displayed in the front panel and the values obtained from the saved traces in the SD card in the majority of the cases. There are no user functions to modify the data points. These stored values had jumps back and forth in time ( *x* axis), even showing some *negative* time points ( Fig. 11b and c ). The y-axis displays the time between individual drops. On Fig. 11b , one can see that no drops were recorded between 1 and 316, then rapid dropping is established. We also noted that the numbers along the x-axis are not in seconds, but an unknown fraction of a second. The manufacturer confirmed that the time points are not in seconds.

#### Drop Size

Due to these inconsistencies, we decided to remove the test tube and measure OT outside the device looking at each drop directly. We observed that drop size was not the same for all tubes but varied quite a bit. This variation in size produced different OT depending on the tube. In a similar manner, we corroborated that LT is measured according to the definition as the time interval between the first drop to fall after the “thrombi stabilization period” and OT. Thus, the value for LT value also depended on the drop size and was ultimately tube dependent.

#### GTT-3 Pressure Mode

When the device was used in GTT-3 or hyper-shear mode, the test tube was connected by a metal needle, through silicone tubing, to an air-pump through a flow/pressure-adjusting bleeding valve. The test tube is then a closed chamber and can only vent through the silicone tube to the machine. The injection pressure can escape only through the valve and the idle motor, both inside the instrument, relatively far from the test tube. As such, any extra pressure can escape from the system much more slowly, compared with test tube with a vent hole, which makes injection of blood different from the GTT-2 mode.

We attempted to use the device in GTT-3 mode but were not able to obtain consistent results. One of the channels lifted the entire tube during the pressure cycle. The pressure cycle was not specified, the tube once sucked blood back into the machine, and the manufacturer later told us to adjust the length of the tubes shipped with the machine as one tube was 3 mm longer than the others. The manufacturer stated that the length of the silicon tubing is critical to avoid any malfunction during the test, particularly to avoid lifting of the syringe after blood injection to happen. We fixed the length; nevertheless, results did not improve. Because of this, we decided to use the device only in GTT-2 mode for all our measurements. We do not know if our device was broken for the GTT-3 mode, or if it is simply an unstable feature.

## Discussion

Our experiments indicate that occlusion in the GTT is dominated by coagulation and the clots are fibrin rich. Coagulation red clots were consecutively shown by (1) histological staining of fibrin, (2) an OT longer than SIPA, (3) significant lengthening of OT by adding anticoagulants, and (4) a shortening of LT by adding tPA. Coagulation induced by Virchow's triad would require a stagnant or low shear rate zone, which exists between and after the beads where the red clots formed. Thus, the formation of blood clot at a long OT is consistent with a mechanism of the coagulation cascade rather than the faster SIPA. We demonstrated that an alternative mechanism of occlusion by SIPA could be induced by modifying the test section, where SIPA was characterized by (1) histological staining of platelets, (2) more rapid occlusion (shorter OT), (3) relative resistance to anticoagulation, and (4) a long LT even with tPA.

The mean OT of 526 seconds in our version of the GTT was similar to the OT of 495 seconds by Suehiro et al, 32 481 seconds by Yamamoto et al, 14 and 524.9 seconds by Otsui et al 33 that were published after 2010. This OT appears to be much longer than reported values using earlier versions of GTT from 2003 and 2006 ( Table 2 ). Unfortunately, comparisons between papers are obscured as the GTT has been produced with different gaps and beads that are not always reported. At times, the GTT had two, three, and four gaps. Similarly, the bead has been variously made of steel and a ceramic. It is unknown when and what changes were made over the years to both the cartridge and the instrument. We do not know the dimensions of the gaps, nor the size of the opening to produce the droplets. These dimensions could have a strong influence on the measured OT between studies.

The intrasample variability in our study was 15% ( *n* = 7) and an interindividual variation of 36% ( *n* = 7). Our intra-assay CV was higher than the intra-assay reported by Rosser et al 34 of 6% ( *n* = 1) and interindividual variation of 27% ( *n* = 32) reported by Yamamoto et al. 14 A large interindividual variation (i.e., large false positive and false negative values) would reduce the ability of this test to distinguish normal versus abnormal clinical populations.

Point-of-care devices are meant to be used at the location of blood draws. Citrate or heparin might be used to extend the time-to-testing. Unfortunately, heparin effectively destroyed the OT for the GTT that verifies previous reports that anticoagulants should not be used with this test. A previous study also reported occlusion suppression (OT >1,000) by using citrate and ethylenediaminetetraacetic acid (EDTA). 35 In contrast, heparin and citrate had a smaller effect on the mGTT, likely due to the SIPA mechanism of occlusion. Both heparin and citrate treated blood led to OTs in the mGTT that might be correlated back to the untreated blood values, since the variance was not high.

The importance of surface (steel, ceramic, glass, and collagen) on the performance of the GTT was evident. The GTT system changes in bead material, gap size, gap number, shear rate, user injection rate, and/or problems in the electronic readouts are not described by the manufacturer. Care should be taken to *not* rely on results from outdated versions of the GTT as representative of newer versions with system changes.

We came to appreciate that the system-defined LT only requires a single drop to emanate from the tip of the test tube. As the drop grows slowly, the drop size and detachment were highly variable and influenced greatly by tube, and a “skin” holding the last drop. The company describes LT as “endogenous lysis” suggesting reperfusion of an end organ. We never witnessed the running of normal blood flow at LT without tPA. An alternative explanation may be that serum continues to seep through the permeable clot and collects as a drop. Such thrombus permeability has been described and quantified for fibrin and platelet-rich thrombi. Consequently, LT may be related to clot structure rather than endogenous lysis.

We experienced some limitations with the device. The GTT device we evaluated was purchased directly from the manufacturer in December 2018 and was used according to all provided instructions. Our struggles with injection rate early in the testing may reflect our inexperience rather than any issues with the machine. Thus, we have excluded any results from the first 6 months of use for this reason. GTT timing on the chip is not in seconds, but some value less than a second since 10 minutes yielded many more than 900 time points. The printout has spurious results. The pressure tubes in our unit, and thus GTT-3, did not work. The manufacturer's manual does not specify whether there are two, three or four gaps in between beads and tube walls. Thus, we used a microCT to establish that our test tube had three gaps of different size. Apparently, the manufacturer has changed the beads from steel to ceramic, but it is unknown when this took place and if the OT values from prior iterations are consistent with current devices. The drop size was highly variable by injection speed, tube, and over time. Additionally, it is unknown if normal ranges are the same as described by the manufacturer or need to be calibrated to each center like PT/PTT. The definition of LT is defined as the time for a single new drop to appear. The interpretation of LT may reflect endogenous plasminogen levels, thrombus permeability, or something else.

## Conclusion

GTT is a point-of-care device designed to test for thrombi from untreated blood. We explored the behavior of the GTT in terms of the possible different mechanisms of thrombosis: coagulation or SIPA. GTT develops a red clot that is rich in fibrin, occludes in approximately 8 minutes consistent with the kinetics of the coagulation cascade, has a low shear zone and an artificial surface needed for Virchow's triad, and is affected strongly by heparin. The LT could be shortened by tPA. These results consistently point to occlusion by a coagulation mechanism, despite the existence of a high shear gap in the system. GTT could be modified to induce SIPA with a platelet-rich thrombus, that occluded much faster over collagen, and was relatively resistant to heparin or tPA. The evidence points to the GTT as occluding primarily due to fibrin-rich red clot from coagulation rather than high shear platelet aggregation and occlusion associated with arterial thrombosis.

## Notes

**‘What is Known on This Topic’**

- The global thrombosis test (GTT) is a point of care device that tests thrombotic and thrombolytic status from untreated blood.
- The end points to evaluate thrombotic and thrombolytic status are occlusion time and lysis time, respectively.
- The test is performed exposing untreated blood to flow in a zone that produces both high and low shear stress, where clots are formed.

**‘What Does This Paper Add?’**

- We demonstrated that the GTT occludes primarily due to fibrin-rich red clot from coagulation rather than high shear-induced platelet aggregation (SIPA) and occlusion associated with arterial thrombosis.
- We demonstrated that an alternative mechanism of SIPA or white clot might be induced in the GTT by modifying the device.
- Coagulation as red clots were shown by histology, an occlusion time longer than SIPA, significant lengthening of occlusion time by adding anticoagulants typically used in clinics (heparin and citrate), and a shortening of lysis time by adding tissue plasminogen activator human, tPA.

## References

[1] Principle & technical details—How GTT worksAccessed July 18, 2020 at:https://www.thromboquest.com/newpage

[2] Home—Global Thrombosis TestAccessed July 18, 2020 at:https://www.thromboquest.com/

[3] Cadroy Y Horbett T A Hanson S R Discrimination between platelet-mediated and coagulation-mediated mechanisms in a model of complex thrombus formation in vivo J Lab Clin Med 1989 113 04 436 448 2522978

[4] Dittman W A Majerus P W Structure and function of thrombomodulin: a natural anticoagulant Blood 1990 75 02 329 336 2153035

[5] Hoffman M Monroe D M III A cell-based model of hemostasis Thromb Haemost 2001 85 06 958 965 11434702

[6] Jesty J Nemerson Y The pathways of blood coagulation 5 ^th^ ed. McGraw-Hill 1995 1222 1238 https://accessmedicine.mhmedical.com/content.aspx?bookid=2962&sectionid=253249871

[7] Mann K G Nesheim M E Church W R Haley P Krishnaswamy S Surface-dependent reactions of the vitamin K-dependent enzyme complexes Blood 1990 76 01 1 16 2194585

[8] Casa L DC Ku D N Thrombus formation at high shear rates Annu Rev Biomed Eng 2017 19 01 415 433 28441034 10.1146/annurev-bioeng-071516-044539

[9] Kim D Bresette C Liu Z Ku D N Occlusive thrombosis in arteries APL Bioeng 2019 3 04 41502 10.1063/1.5115554 PMC6863762 31768485

[10] Jackson S P The growing complexity of platelet aggregation Blood 2007 109 12 5087 5095 17311994 10.1182/blood-2006-12-027698

[11] Bark D L Jr Ku D N Wall shear over high degree stenoses pertinent to atherothrombosis J Biomech 2010 43 15 2970 2977 20728892 10.1016/j.jbiomech.2010.07.011

[12] Ku D N Flannery C J Development of a flow-through system to create occluding thrombus Biorheology 2007 44 04 273 284 18094451

[13] Carstairs K C The identification of platelets and platelet antigens in histological sections J Pathol Bacteriol 1965 90 01 225 231 5320852 10.1002/path.1700900124

[14] Yamamoto J Inoue N Otsui K Ishii H Gorog D A Global Thrombosis Test (GTT) can detect major determinants of haemostasis including platelet reactivity, endogenous fibrinolytic and thrombin generating potential Thromb Res 2014 133 05 919 926 24613697 10.1016/j.thromres.2014.02.018

[15] Tamura N Kitajima I Kawamura Y Important regulatory role of activated platelet-derived procoagulant activity in the propagation of thrombi formed under arterial blood flow conditions Circ J 2009 73 03 540 548 19179771 10.1253/circj.cj-08-0465

[16] Saraf S Christopoulos C Salha I B Stott D J Gorog D A Impaired endogenous thrombolysis in acute coronary syndrome patients predicts cardiovascular death and nonfatal myocardial infarction J Am Coll Cardiol 2010 55 19 2107 2115 20447533 10.1016/j.jacc.2010.01.033

[17] Spinthakis N Farag M Gue Y X Srinivasan M Wellsted D M Gorog D A Effect of P2Y _12_ inhibitors on thrombus stability and endogenous fibrinolysis Thromb Res 2019 173 102 108 30500673 10.1016/j.thromres.2018.11.023

[18] Casa L DC Deaton D H Ku D N Role of high shear rate in thrombosis J Vasc Surg 2015 61 04 1068 1080 25704412 10.1016/j.jvs.2014.12.050

[19] Johar R S Smith R P Assessing gravimetric estimation of intraoperative blood loss J Gynecol Surg 1993 9 03 151 154 10171989 10.1089/gyn.1993.9.151

[20] Mehrabadi M Casa L DC Aidun C K Ku D N A predictive model of high shear thrombus growth Ann Biomed Eng 2016 44 08 2339 2350 26795978 10.1007/s10439-016-1550-5

[21] Movat H Z Demonstration of all connective tissue elements in a single section; pentachrome stains AMA Arch Pathol 1955 60 03 289 295 13248341

[22] Roman N Perkins S F Perkins E M Jr Dolnick E H Orcein-hematoxylin in iodized ferric chloride as a stain for elastic fibers, with metanil yellow counterstaining Stain Technol 1967 42 04 199 202 4166561 10.3109/10520296709115008

[23] Para A Bark D Lin A Ku D Rapid platelet accumulation leading to thrombotic occlusion Ann Biomed Eng 2011 39 07 1961 1971 21424850 10.1007/s10439-011-0296-3

[24] Para A N Ku D N A low-volume, single pass in-vitro system of high shear thrombosis in a stenosis Thromb Res 2013 131 05 418 424 23535566 10.1016/j.thromres.2013.02.018

[25] Kim D A Ashworth K J Di Paola J Ku D N Platelet α-granules are required for occlusive high-shear-rate thrombosis Blood Adv 2020 4 14 3258 3267 32697818 10.1182/bloodadvances.2020002117 PMC7391145

[26] Neeves K B Onasoga A A Hansen R R Sources of variability in platelet accumulation on type 1 fibrillar collagen in microfluidic flow assays PLoS One 2013 8 01 e54680 23355889 10.1371/journal.pone.0054680 PMC3552855

[27] Griffin M T Kim D Ku D N Shear-induced platelet aggregation: 3D-grayscale microfluidics for repeatable and localized occlusive thrombosis Biomicrofluidics 2019 13 05 54106 10.1063/1.5113508 PMC6773594 31592301

[28] Gorog D Becker R Point-of-care platelet function tests: relevance to arterial thrombosis and opportunities for improvement J Thromb Thrombolysis 2021 51 1 11 32529549 10.1007/s11239-020-02170-z PMC7829242

[29] Siedlecki C A Hemocompatibility of Biomaterials for Clinical Applications Elsevier 2018 https://doi.org/10.1016/C2014-0-04140-8(https://www.elsevier.com/books/hemocompatibility-of-biomaterials-for-clinical-applications/siedlecki/978-0-08-100497-5)

[30] Taomoto K Ohnishi H Kuga Y Platelet function and spontaneous thrombolytic activity of patients with cerebral infarction assessed by the global thrombosis test Pathophysiol Haemost Thromb 2010–1037 01 43 48 20516672 10.1159/000315494

[31] Suehiro A Wakabayashi I Uchida K Yamashita T Yamamoto J Impaired spontaneous thrombolytic activity measured by global thrombosis test in males with metabolic syndrome Thromb Res 2012 129 04 499 501 21752433 10.1016/j.thromres.2011.06.019

[32] Suehiro A Wakabayashi I Yamashita T Yamamoto J Attenuation of spontaneous thrombolytic activity measured by the global thrombosis test in male habitual smokers J Thromb Thrombolysis 2014 37 04 414 418 23842702 10.1007/s11239-013-0962-4

[33] Otsui K Gorog D A Yamamoto J Global Thrombosis Test—a possible monitoring system for the effects and safety of dabigatran Thromb J 2015 13 01 39 26648789 10.1186/s12959-015-0069-6 PMC4672538

[34] Rosser G Tricoci P Morrow D PAR-1 antagonist vorapaxar favorably improves global thrombotic status in patients with coronary disease J Thromb Thrombolysis 2014 38 04 423 429 24676931 10.1007/s11239-014-1075-4

[35] Yamamoto J Yamashita T Ikarugi H Görög Thrombosis Test: a global in-vitro test of platelet function and thrombolysis Blood Coagul Fibrinolysis 2003 14 01 31 39 12544726 10.1097/01.mbc.0000046170.06450.9b

[36] Ikarugi H Yamashita T Aoki R Ishii H Kanki K Yamamoto J Impaired spontaneous thrombolytic activity in elderly and in habitual smokers, as measured by a new global thrombosis test Blood Coagul Fibrinolysis 2003 14 08 781 784 14614361 10.1097/00001721-200312000-00016

[37] Nishida H Murata M Miyaki K Omae K Watanabe K Ikeda Y Gorog Thrombosis Test: analysis of factors influencing occlusive thrombus formation Blood Coagul Fibrinolysis 2006 17 03 203 207 16575258 10.1097/01.mbc.0000220242.22714.b3

[38] Saraf S Wellsted D Sharma S Gorog D A Shear-induced global thrombosis test of native blood: pivotal role of ADP allows monitoring of P2Y12 antagonist therapy Thromb Res 2009 124 04 447 451 19476973 10.1016/j.thromres.2009.04.013

